# Prevalence and risk factors for mast cell tumours in dogs in England

**DOI:** 10.1186/2052-6687-2-1

**Published:** 2015-01-26

**Authors:** Stephanie JW Shoop, Stephanie Marlow, David B Church, Kate English, Paul D McGreevy, Anneliese J Stell, Peter C Thomson, Dan G O’Neill, David C Brodbelt

**Affiliations:** Arthritis Research UK Centre for Epidemiology, Institute of Inflammation and Repair, University of Manchester, Oxford Road, Manchester, M13 9PT England; Veterinary Epidemiology, Economics and Population Health, The Royal Veterinary College, Hawkshead Lane, North Mymms, Hatfield Herts, AL9 7TA England; Small Animal Medicine and Surgery Group, The Royal Veterinary College, Hawkshead Lane, North Mymms, Hatfield Herts, AL9 7TA England; Pathology and Pathogen Biology, The Royal Veterinary College, Hawkshead Lane, North Mymms, Hatfield Herts, AL9 7TA England; R.M.C. Gunn Building (B19), Faculty of Veterinary Science, The University of Sydney, Sydney, NSW 2006 Australia

**Keywords:** Mastocytoma, Mast cell, Canine, Prevalence, Primary-care practice, Epidemiology, Oncology

## Abstract

**Background:**

Mast cell tumour (MCT) appears to be a frequent tumour type in dogs, though there is little published in relation to its frequency in dogs in the UK. The current study aimed to investigate prevalence and risk factors for MCTs in dogs attending English primary-care veterinary practices.

**Methods:**

Electronic patient records from practices participating in the VetCompass animal surveillance project between July 2007 and June 2013 were searched for MCT diagnosis. Various search terms and standard diagnostic terms (VeNom codes) identified records containing MCT diagnoses, which were evaluated against clinical criteria for inclusion to the study. MCT prevalence for the entire dataset and specific breed types were calculated. Descriptive statistics characterised MCT cases and multivariable logistic regression methods evaluated risk factors for association with MCT (P < 0.05).

**Results:**

Within a population of 168,636 dogs, 453 had MCT, yielding a prevalence of 0.27% (95% confidence interval (CI) 0.24% - 0.29%). The highest breed type specific prevalences were for the Boxer at 1.95% (95% CI 1.40% - 2.51%), Golden Retriever at 1.39% (0.98% - 1.81%) and Weimaraner at 0.85% (95% CI 0.17% to 1.53%). Age, insurance status, neuter status, weight and breed type were associated with MCT diagnosis. Of dogs of specific breed type, the Boxer, Pug and Staffordshire Bull Terrier showed greater odds of MCT diagnosis compared with crossbred dogs. Conversely, the German Shepherd Dog, Border Collie, West Highland White Terrier, Springer Spaniel and Cocker Spaniel had reduced odds of MCT diagnosis compared with crossbred dogs. No association was found between MCT diagnosis and sex.

**Clinical significance:**

This study highlights a clinically significant prevalence of MCT and identifies specific breed types with predisposition to MCT, potentially aiding veterinarian awareness and facilitating diagnosis.

## Lay summary

Mast cell tumour (MCT) is the most common skin tumour type in dogs, but little is known about its frequency in the general dog population nor its frequency in particular dog breeds in the UK. This study has investigated the frequency of the disease, and possible risk factors associated with the development of MCT.

This has been conducted by analysing the large number of electronic patient health records contained within the VetCompass animal surveillance project collected between July 2007 and June 2013.

Data were available on a population of 168,636 dogs and within this 453 (0.27%) had MCT. The breeds with the highest breed specific prevalences were Boxer, Golden Retriever and Weimaraner. Conversely, some breeds appeared to be protected.

Age, insurance status, neuter status, weight and breed type were associated with MCT diagnosis. No association was found between MCT diagnosis and gender.

Such information helps to increase awareness of this condition, particularly in certain breeds, to owners and veterinarians.

## Background

Mast cell tumours (MCTs) are a frequent tumour type in dogs [1], accounting for 7-21% of cutaneous tumours diagnosed [1–4]. Dobson and others (2002) reported the incidence of MCT in English dogs as 129 in 100,000 dogs per year [5]; however, this study was limited to insured animals and thus may generalise poorly to the wider population of dogs in England. MCTs occur primarily in the dermis and subcutaneous tissues and can be confirmed in 92-96% of cases through fine needle aspirate (FNA) cytology [6]. Histopathology allows description of the degree of MCT malignancy and clinical grading [6] using two grading systems. Using the Patnaik system, MCTs are categorised into three grades (I, II & III), the third being more clinically important because tumours of this grade are often more aggressive and may metastasise [7]. The more recently proposed Kiupel two tier system categorises MCTs into high or low-grade in order to increase concordance among veterinary pathologists [8].

There are likely to be many factors contributing to the development of MCT which may be genetically influenced [6]. Up to 40% of dogs with subcutaneous and cutaneous MCTs have been found to have mutations in a proto-oncogene, *c-kit*[6]. Other potential factors include neuter status, sex, age, weight, insurance status and specific breed types. Females [9], and particularly neutered females [10] have been associated with increased risk of MCT in some studies, although other studies failed to identify an association between sex and MCT [11, 12]. While the mean age at presentation of cutaneous MCTs has been reported at between 7.5 and 9 years old [9], one study found poorly differentiated (grade III) tumours more common in younger dogs, although the study population consisted of only one breed, the Shar-Pei [13]. Likewise, while one study found no difference in pre-disposition to MCTs between dogs of specific breed types and mixed breed (crossbred) dogs overall [14], certain breed types have previously been associated with this condition. Most commonly, Boxers and Golden Retrievers [10, 15, 16] and other larger breed types have been associated with pre-disposition to MCT whereas smaller breed types are reported to be at lower risk of the condition [10]. Less commonly, breed types including Pugs [10, 17], Weimaraners [15, 16] and the mastiff and terrier phylogenetic clusters [10] have also been associated with having MCTs.

This study aimed to estimate the prevalence and explore risk-factors for the development of MCTs in dogs, using a large sample of animals attending primary-care veterinary practices in England. By identifying factors associated with a higher risk of developing MCTs, it was hoped to improve the index of suspicion for this disease and hence early recognition of this important condition.

## Results

The VetCompass system documented 168,636 dogs from a total of 94 veterinary practices within England between 28^th^ June 2007 and 30^th^ June 2013. Of these, 453 dogs met the clinical criteria for inclusion to the study and were classified as dogs presenting with MCT at a participating veterinary practice during the study period. Thus, the MCT prevalence was estimated at 0.27% (95% CI 0.24% to 0.29%) over the study period. Individual breed type MCT prevalences that exceeded this overall estimate included that of the Boxer at 1.95% (95% CI 1.40% to 2.41%), the Golden Retriever at 1.39% (95% CI 0.98% to 1.81%), the Weimaraner at 0.85% (95% CI 0.17% to 1.53%), the Labrador Retriever at 0.72% (95% CI 0.58% to 0.85%), the Staffordshire Bull Terrier at 0.51% (95% CI 0.39% to 0.62%) and the Pug at 0.50% (95% CI 0.13% to 0.88%). Individual breed-type MCT prevalences that fell below the overall estimate included the Springer Spaniel at 0.20% (95% CI 0.06% to 0.35%), the Jack Russell Terrier at 0.16% (95% CI 0.09% to 0.23%), the West Highland White Terrier at 0.07% (95% CI 0.00% to 0.15%), the Border Collie at 0.07% (95% CI 0.00% to 0.14%), the Cocker Spaniel at 0.06% (95% CI 0.00% to 0.12%), the Yorkshire Terrier at 0.04% (95% CI 0.00% to 0.09%) and the German Shepherd Dog at 0.02% (95% CI 0.00% to 0.05) (Table 1A). Overall MCT prevalence for dogs of specific breed types was 0.29% (95% CI 0.26 – 0.32%) and overall crossbred prevalence was 0.18% (95% CI 0.14 – 0.22) (Table 1B).

**Table 1:** **Breed-type specific prevalence of mast cell tumour (MCT) diagnosis with 95% confidence intervals (CI)**

**A) Specific breed types**				
**Breed-type**	**Cases (n)**	**Total (n)**	**MCT prevalence (%)**	**95% CI (%)**
Boxer	47	2406	1.95	1.40 - 2.51
Golden Retriever	43	3086	1.39	0.98 - 1.81
Weimaraner	6	705	0.85	0.17 - 1.53
Labrador Retriever	106	14781	0.72	0.58 - 0.85
Staffordshire Bull Terrier	72	14219	0.51	0.39 - 0.62
Pug	7	1391	0.50	0.13 - 0.88
Springer Spaniel	8	3906	0.20	0.06 - 0.35
Jack Russell Terrier	18	11333	0.16	0.09 - 0.23
West Highland White Terrier	3	4254	0.07	0.00 - 0.15
Border Collie	3	4501	0.07	0.00 - 0.14
Cocker Spaniel	4	6353	0.06	0.00 - 0.12
Yorkshire Terrier	2	5512	0.04	0.00 - 0.09
German Shepherd Dog	1	5993	0.02	0.00 - 0.05
**B) Summary results**				
Specific breed type	388	132139	0.29	0.26 – 0.32
Crossbred	65	36361	0.18	0.14 - 0.22
Unknown breed	0	136	-	-
Total	453	168636	0.27	0.24 – 0.29

Dogs were recruited from a VetCompass population of 168,636 dogs. Breed types are listed from highest to lowest prevalence.

Of the cases, 48% were male (n =218), 64% insured (n =291) and 71% neutered (n =323). The median age at diagnosis was 8.2 years (interquartile range (IQR) 6.0 to 10.2 yr) and median weight 27.7 kg (IQR 20.0 to 34.2 kg).The most common breed types affected were the Labrador Retriever (23%, n =106), Staffordshire Bull Terrier (16%, n =72), Boxer (10%, n =47), Golden Retriever (9%, n =43), Jack Russell Terrier (4%, n =18), Springer Spaniel (2%, n =8), Pug (2%, n =7) and Weimaraner (1%, n =6). Fourteen percent were crossbred (n =65). Of the controls, 52% were male (n =1,067), 26% insured (n =527) and 40% neutered (n =820). The median age was 3.1 years (IQR 0.5 to 7.2 yrs) and median weight 11.6 kg (IQR 6.0 to 24.7 kg). The most common control breed types were the Labrador Retriever (11%, n =215), Staffordshire Bull Terrier (8%, n =159), Jack Russell Terrier (8%, n =155), Cocker Spaniel (4%, n =86), West Highland White Terrier (3%, n =66), German Shepherd Dog (3%, n =63), Border Collie (3%, n =60), Yorkshire Terrier (3%, n =57), Springer Spaniel (2%, n =50) and Golden Retriever (2%, n =39). Nineteen percent were crossbred (n =391). The 453 cases and 2,036 controls (1:4 case to control ratio) were taken forward for evaluation of risk factors for a diagnosis of MCT.

### Risk factor analysis

**Figure 1:**
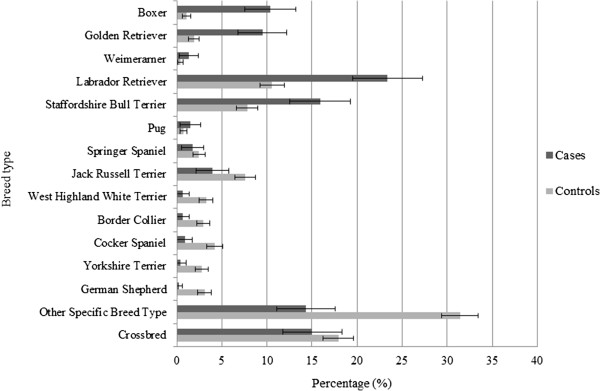
**Percentage of mast cell tumour (MCT) positive and non-MCT dogs attributed to specific breeds.** 95% confidence intervals are displayed relating to these values. Cases (n = 453) and controls (n = 2036) were selected from a VetCompass population of 168,636 dogs.

Univariable analysis identified insured and entire dogs as having significantly increased odds of MCT. Weight and age were also associated with MCT, with the categories at highest odds being 30.01 to 40.00 kg, compared to ≤10.00 kg and 8 to 10 years of age, compared to ≤2.00 years, respectively (Table 2). In addition, the proportion of certain breed types in case and control groups differed (Figure 1), suggesting at least a univariate association between specific breed type and MCT diagnosis. In particular, the Labrador Retriever, Staffordshire Bull Terrier, Golden Retriever, Boxer, Pug and Weimaraner appeared to be more frequently represented among cases than controls. There was also a significantly larger proportion of specific breed types compared to crossbreds in case compared to control samples. There was no significant difference in sex between the two groups (Table 2).

**Table 2:** **Risk factors for mast cell tumour (MCT) from univariable analysis**

Variable	Category	Case	Control	OR	95% CI	P-value
Age	≤2.0 years	8	840	1(base)		<0.001
	2.0-4.0 years	22	301	7.7	3.4-17.4	<0.001
	4.0-6.0 years	60	245	25.7	12.1-54.5	0.038
	6.0-8.0 years	82	199	43.3	20.-90.9	<0.001
	8.0-10.0 years	95	171	58.3	27.8-122.3	<0.001
	>10.0 years	108	267	42.5	20.4-88.2	<0.001
Age (continuous)	Median (IQR) (years)	8.2 (6.0 - 10.2)	3.1 (0.5 – 7.2	1.2	1.2-1.3	<0.001
Breed Type	Crossbred	65	391	1(base)		0.016
	Boxer	47	22	12.9	7.3-22.7	<0.001
	Golden Retriever	43	39	6.6	4.0-11.0	<0.001
	Weimaraner	6	9	4.0	1.4-11.6	0.028
	Labrador Retriever	106	215	3.0	2.1-4.2	<0.001
	Pug	7	15	2.8	1.1-7.2	0.096
	Staffordshire Bull Terrier	72	159	2.7	1.9-4.0	<0.001
	Springer Spaniel	8	50	1.0	0.4-2.1	0.379
	Jack Russell Terrier	18	155	0.7	0.4-1.2	0.006
	Border Collie	3	60	0.3	0.1-1.0	0.005
	Cocker Spaniel	4	86	0.3	0.1-0.8	0.001
	West Highland White Terrier	3	66	0.3	0.1-0.9	0.002
	Yorkshire Terrier	2	57	0.2	0.1-0.9	0.003
	German Shepherd Dog	1	63	0.1	0.0-0.7	<0.001
	Other specific breed type	68	649	0.6	0.4-0.9	0.456
Purebred status	Crossbred	65	391	1(base)		
	Of breed type	388	1654	1.4	1.1-1.9	0.016
Insurance status	Not insured	162	1502	1(base)		
	Insured	291	527	5.1	4.1-6.4	<0.001
Neuter status	Entire	130	102	1(base)		
	Neutered	323	820	0.3	0.2-0.4	<0.001
Sex	Female	233	956	1(base)		
	Male	218	1067	1.2	1.0-1.5	0.090
Weight	≤10.0 kg	49	771	1(base)		<0.001
	10.0-20.0 kg	60	379	2.5	1.7-3.7	<0.001
	20.0-30.0 kg	145	296	7.7	5.4-10.9	<0.001
	30.0-40.0 kg	135	197	10.8	7.5-15.5	<0.001
	>40.0 kg	46	79	9.2	5.8-14.6	<0.001
Weight (continuous) (kg)	Median (IQR)	27.7 (20.0 - 34.2)	11.6 (6.0 – 24.7)	1.1	1.1-1.1	<0.001

Odds ratios (OR) with 95% confidence intervals (CI) are reported. Cases (n = 453) and controls (n = 2036) were selected from a VetCompass population of 168,636 dogs.

All variables tested showed at least a general trend (P ≤0.25) of association with the outcome (Table 2), and were taken forward for evaluation in the multivariable analysis [18] (Table 3). The multivariable model identified age, insurance status, weight, neuter status and specific breeds as statistically significantly associated with MCT diagnosis. Sex was not significantly associated with MCT diagnosis (P =0.18).

**Table 3:** **Risk factors for mast cell tumour (MCT) from final multivariable binary logistic regression model**

Variable	Category	Case	Control	OR	95% CI	P value
Age	≤2.0 years	8	840	1(base)		<0.001
	2.0-4.0 years	22	301	5.7	2.1-15.2	0.001
	4.0-6.0 years	60	245	15.9	6.2-40.4	<0.001
	6.0-8.0 years	82	199	18.3	7.3-45.9	<0.001
	8.0-10.0 years	95	171	38.3	15.1-97.4	<0.001
	>10.0 years	108	267	41.3	16.7-102.5	<0.001
Breed type	Crossbred	65	391	1(base)		<0.001
	Boxer	47	22	10.7	3.7-30.4	<0.001
	Pug	7	15	10.0	1.5-64.5	0.016
	Staffordshire Bull terrier	72	159	4.2	2.2-8.2	<0.001
	Golden Retriever	43	39	2.0	0.9-4.5	0.080
	Weimaraner	6	9	1.4	0.2-9.8	0.718
	Labrador Retriever	106	215	1.3	0.8-2.3	0.319
	Jack Russell Terrier	18	155	0.9	0.4-2.1	0.790
	Cocker Spaniel	4	86	0.3	0.1-0.9	0.037
	Springer Spaniel	8	50	0.3	0.1-0.9	0.029
	West Highland White Terrier	3	66	0.2	0.0-0.8	0.030
	Border Collie	3	60	0.1	0.0-0.5	0.004
	Yorkshire Terrier	2	57	0.1	0.0-1.0	0.053
	German Shepherd Dog	1	63	0.0	0.0-0.4	0.007
	Other specific breed type	68	649	0.4	0.3-0.7	0.001
Insurance Status	Not Insured	162	1502	1(base)		
	Insured	291	527	3.1	2.2-4.5	<0.001
Neuter Status	Entire	130	102	1(base)		
	Neutered	323	820	0.1	0.1-0.2	<0.001
Weight	≤10.0 kg	49	771	1 (base)		0.004
	10.0-20.0 kg	60	379	1.1	0.6-2.1	0.744
	20.0-30.0 kg	145	296	2.6	1.4-4.8	0.002
	30.0-40.0 kg	135	197	2.5	1.3-4.9	0.007
	>40.0 kg	46	79	2.6	1.2-5.6	0.017

Odds ratios (OR) with 95% confidence intervals (CI) are reported. Cases (n = 453) and controls (n = 2036) were selected from a VetCompass population of 168,636 dogs.

**Figure 2:**
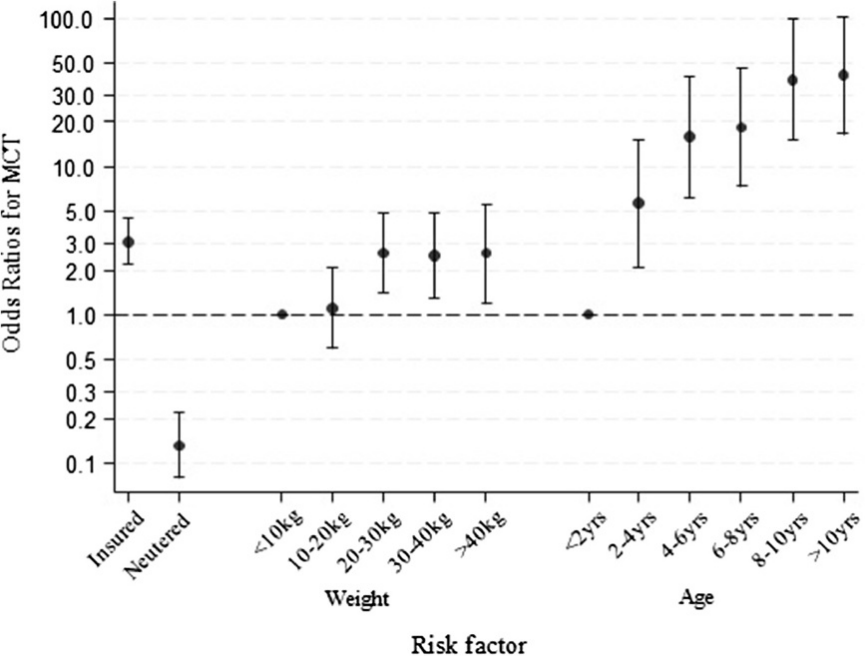
**Risk factors for mast cell tumour (MCT) from final multivariable binary logistic regression model.** Odds ratios for MCT with corresponding 95% confidence intervals are reported. Dogs weighing less than 10 kg and aged less than 2 yrs have odds ratios of 1 as were used as baseline categories for weight and age variables respectively.

Dogs over 10 years old had 41.3 times the odds of MCT diagnosis compared with dogs under 2 years old (95% CI 16.7 to 102.5). Dogs of weight 20 to 30 kg had 2.6 times the odds of MCT diagnosis compared with those under 10 kg (95% CI 1.4 to 4.8). Insured dogs were at 3.1 times the odds (95% CI 2.2 to 4.5) and neutered dogs were at 0.1 times the odds of having MCT (95% CI 0.1 to 0.2) compared to uninsured and entire dogs respectively (Figure 2). Of specific breed types, the Boxer was at 10.7 times (95% CI 3.7 to 30.4), Pug at 10.0 times (95% CI 1.5 to 64.5) and Staffordshire Bull Terrier at 4.2 times the odds (95% CI 2.2 to 8.2) of having MCT compared with crossbred dogs. Conversely, the German Shepherd Dog was at 0.0 times (95% CI 0.0 to 0.4), Border Collie at 0.1 times (95% CI 0.0 to 0.5), West Highland White Terrier at 0.2 times (95% CI 0.0 to 0.8), Springer Spaniel at 0.3 times (95% CI 0.1 to 0.9), and Cocker Spaniel at 0.3 times the odds (95% CI 0.1 to 0.9) of having MCT compared with crossbred dogs. No association was found between sex and MCT diagnosis. Dogs of all other specific breed types other than those individually analysed were at 0.4 times the odds of having MCT than crossbreds (95% CI 0.3-0.0.7). When comparing all specific breed types versus all crossbreds without testing for individual breed effects in the multivariable model, no significant difference in MCT predisposition was observed (P =0.762). A significant interaction was observed between weight and the specific breed type variable, but a likelihood ratio test suggested no significant improvement to model fit (P >0.5), so this interaction was not retained. No other interactions were retained in the final model. Good final model fit was suggested by a Hosmer-Lemeshow test (P =0.831). Clustering at the clinic level was evaluated with clinic ID entered as a random effect but was not statistically significant and therefore was not retained (P =0.49).

## Discussion

This study identified a prevalence of MCT of 0.27% (95% CI 0.24% to 0.29%) over the study period and demonstrated associations between age, insurance status and specific breed types with MCT diagnosis. Highest MCT breed type prevalences were identified for the Boxer at 1.95% (95% CI 1.40% - 2.51%), Golden Retriever at 1.39% (0.98% - 1.81%) and Weimaraner at 0.85% (95% CI 0.17% to 1.53%). A previous study estimated an incidence of MCT among insured UK dogs of 129 in 100,000 dogs per year (95% CI 107 to 145) [5]. However, as this study’s population was limited to insured animals, the results may not generalise well to the overall dog population. Insured dogs are more likely to be younger [5], as policies are often cancelled in older dogs [19] and more likely to be of specific breed-types [20]. Insured dogs are also more likely to undergo further diagnostic testing and treatments [21] compared with uninsured dogs, which potentially led to an overestimation of MCT incidence by Dobson and others [5]. Conversely, insurance data may underestimate true incidence of MCT as only cases that are not excluded and whereby the deductible excess does not exceed the cost of diagnosis and treatment are included [21]. Therefore, the estimate reported in the current study is more generalisable to practice-attending dogs and to the general canine population in England. A Dutch study estimated incidence at 265 in 100,000 dog-years and focused purely on Labrador Retrievers [22]. It is likely that the latter’s incidence estimation was much larger due to the potential predisposition of Labrador Retrievers to purely cutaneous MCTs [10, 23, 24]. Whilst Labrador Retrievers were not found to have a significant association with MCT diagnosis in the current study, it may be that this breed type is associated with only cutaneous MCTs or the current finding may highlight differences between study populations. Incidence was not calculated in the current study as it was not always clear if the cancer cases were newly diagnosed, hence it was considered more reliable to report the frequency of all existing cases in relation to all practice attending dogs during the study period and thus period prevalence. In a large study in the US, Villamil and others (2011) reported 0.25% of 1,139,616 dogs analysed were diagnosed with cutaneous MCT [23], similar to the current study’s estimation.

The current study identified that older dogs were at increased odds of MCT, with those over 10 years being around 41 times greater odds than those under 2 year of age. Previous studies have also found older dogs to be more susceptible to MCTs [23, 25], consistent with the findings of the current study, with the average age for MCT diagnosis in dogs between 7.5-9 years of age [9]. In the current study, the median age of cases at presentation was 8.2 years (IQR 6.0-10.2 yr), compared to that of controls which was 3.1 years (IQR 0.5-7.2 yr). In summary, the current findings suggest that MCTs generally present in older dogs. A study focusing on Shar-Peis identified younger animals (median age 5 years) to be commonly afflicted with high grade MCTs, with 28% of cases under 2 years of age [13]. However, the small sample size (n =18) and single breed make it difficult to generalise the results from this earlier study.

Insured dogs had a higher odds of MCT compared with the control group in the current analysis. Biological differences between insured and uninsured dogs may have partially accounted for this association, as a higher percentage of dogs of some specific breed types have been reported to be insured compared with crossbreds [21]. Conversely, insurance cover is frequently cancelled in older dogs and so the insured group may have under-represented older dogs that had increased odds of MCT. However, diagnostic testing and treatments likely differed between insured and non-insured dogs. Insured dogs may have been more likely to undergo further tests that confirm MCT [21], and therefore to have met clinical criteria for inclusion to the study.

Similar to Peters and others [11] and Miller [13], the current study found no association between sex and MCT. Consistent with the findings of White and colleagues [10] and Zink and others [26], the current study also reported an association between neuter status and MCT development. However, whilst Zink and others [26] reported increased odds of MCT for neutered Vizslas and White and others [10] reported an increased risk of MCT for neutered females, the current study found decreased odds of MCT for neutered dogs in general. However, limitations of the present study may also contribute to these conflicting findings. In the practice management systems, each dog was assigned one neuter status which was promptly updated without notification of date when this status changed. At the time of the data query, neuter status may have been different from that when initial MCT diagnosis was made, therefore this study may have underestimated the number of dogs with MCT that were entire at diagnosis. Further investigation into a potential association between neuter status in both sexes and MCT diagnosis is merited.

Previous studies describing risk-factors for MCT were mainly limited to specific breed types, focused purely on cutaneous MCTs and had smaller sample sizes than the current study. Larger-sized breeds were previously reported at higher risk of developing MCT than smaller and medium breeds [10]. Similarly, the current study found that dogs over 20 kg had over twice the odds of having MCT compared to those under 10 kg. The current study also supports the findings of a large US study of no difference in MCT diagnosis between dogs of specific breed types and crossbred dogs in general [14]. This is likely a product of increased risk for a few specific breed types and decreased risk for the majority of dogs of other breed types, supported by the significantly decreased MCT odds for the ‘other specific breed type’ category in the present analysis.

The current study supports the general consensus that Boxers have increased risk of MCTs [10, 11, 15, 16, 23, 25, 27]. Consistent with previous work [11, 16, 23], the study also identified Staffordshire Bull Terriers as being at higher odds and it is hypothesised that their predisposition, including that of Boxers and other bull-type breeds, may be linked to common ancestry [11] since these breed types are closely phylogenetically clustered [27]. As also reported by Murphy and others [16], Villamil and others [23], White and others [10] and McNiel and others [17], the current study identified Pugs as having high odds of developing MCTs. Golden Retrievers [10, 15, 16, 23, 27], Labrador Retrievers [10, 23, 24] and Weimaraners [15, 16, 27] have previously been found to be at high risk of cutaneous MCTs. Though the current study reports trends supporting these findings, it did not detect a statistically significant association for the Golden Retriever, Labrador Retriever or the Weimaraner compared to crossbred dogs. However, given the small sample size for Weimaraners (case n =6, control n =9), the current study may have had limited power to explore infrequently presented breed types and smaller magnitudes of association. This study corroborates previous work that found German Shepherd Dogs [23, 27], Cocker Spaniels, West Highland White Terriers and Border Collies to be at decreased odds of MCT [27] compared to crossbred dogs. However, in contrast to previous work this study did not detect a reduction in odds of MCT in Yorkshire Terriers [10, 23].

Some limitations to the current study are worth noting. When assessing common breed types in control samples, a number had small sample sizes in the case group. Border Collies, German Shepherd Dogs, Weimaraners, West Highland White Terriers and Yorkshire Terriers were analysed separately because previous studies suggested protective effects for these breed types [10, 23, 27]. This may be an effect of partial confounding by age. The case group will have had older dogs than the control group as older dogs were at greater odds of MCT and therefore fewer dogs of large breed type, which have been reported to have shorter longevity [28]. However, age of dogs was retained in the final multivariable model and should have, at least in part, adjusted for this association between breed and age structure. Matching cases to controls based on a similar age-structure may have improved interpretation of breed type associations with MCT, but would have prevented investigation into any association between age and this condition. Based on the stated case definition, the prevalence of MCT was likely to be under-estimated. Subcutaneous tumours may be less likely to be diagnosed compared with cutaneous MCTs because of their less prominent nature. In addition, some dogs with MCT will have been excluded from the case sample where a diagnosis of MCT was made but no definitive laboratory results from FNA or histopathology were recorded. This included dogs where a history of MCT only was cited and where a MCT diagnosis was recorded but euthanasia followed without a definitive test. Likewise, in a small number of dogs, an impression smear alone was used to diagnose MCT. Whilst in theory an MCT diagnosis could be obtained from an ulcerated mass through an impression smear, if there is superficial inflammation associated with the lesion, the smear may be difficult to interpret [29]. Conversely, it was possible that controls may have had MCTs, but by excluding dogs with any mention of mast cell in their case notes, this study aimed to minimise the number of misclassified controls. Insurance status of study dogs may not have been completely accurate. Where there were no insurance data at the date of diagnosis for case dogs, the nearest possible previous insurance status was used. Thus, insurance status may have changed without documentation by the date of diagnosis. For controls, the nearest possible insurance status was used as the date of first presentation to a veterinarian.

## Conclusion

This study estimated a clinically relevant prevalence of MCT in dogs attending primary-care practices in England at approximately 0.27% (95% CI 0.24% to 0.29%) and identified associations with older, insured, heavier dogs, and specific breed types with diagnosis. Boxers, Pugs and Staffordshire Bull Terriers were at increased odds of MCT compared to crossbred dogs. Conversely, German Shepherd Dogs, West Highland White Terriers, Border Collies and Cocker Spaniels were at lower odds of MCT. Further investigation is warranted to explore MCT occurrence in less common breed types as well as associations with subcutaneous MCT and malignancy severity.

## Method

The study population was selected from the VetCompass animal surveillance database [30], which documents clinical records from English primary-care veterinary practices. Data relating to clinical consultations between 28^th^ June 2007 and 30^th^ June 2013 were collected from a group of practices using VetCompass predominantly in central and south eastern England. Electronic patient record (EPR) data included a summary diagnosis term selected from a list of standardised veterinary nomenclature (VeNom) codes, developed by the VeNom Coding Group [31], which were selected by clinicians at the time of the ‘episodes of care’. Additional clinical fields collected included unique animal and clinic identification numbers, date of birth, breed type, sex, neuter status, weight, insurance status, episode of care date, clinical notes and details of specific diagnosis, confirmatory testing methods and treatments including any surgical procedures. Institutional ethics approval was gained (*RVC URN 2001 1101*).

The case definition for MCT included evidence of a final diagnosis of MCT recorded either by a veterinarian within the clinical notes or on an insurance claim submission. Additionally, evidence of results from a combination of fine needle aspirate (FNA) cytology and/or biopsy histopathology was required to confirm this diagnosis. Dogs that had confirmatory testing using only impression smears were not included due to difficulty in the interpretation of such smears [29]. Cases were identified from the clinical notes based on this case definition, via the standard diagnosis (VeNom codes) and the clinical free text notes. Search terms applied for the free text clinical notes included ‘mast cell’, ‘mast_’, ‘mct’ and ‘masto’.

Control animals were selected using a random number generator [32] from dogs not diagnosed with MCT. Since minimal increase in statistical power can be gained beyond four controls per case [33], a subset of control animals were selected at a 1:4 case to control ratio [34]. Controls were not matched to cases on any clinical or phenotypical criteria in order to avoid excluding any potential risk factors for MCT from the analysis. Therefore, characteristics of controls derived randomly from the VetCompass database were likely to reflect the general practice attending canine population. These random numbers were then associated with unique patient identification numbers using the VLOOKUP function in Microsoft Excel 2010 [35]. Controls were excluded if they had evidence of suspicion or confirmation of MCT or no evidence of presentation to a veterinary surgeon within the existing case notes. Risk factors tested for association with MCT were age, sex, insurance status, neuter status, weight and breed type.

Prevalence of MCT diagnosis was estimated from the number of cases divided by the population of dogs documented in the VetCompass system throughout the study period. Prevalence of MCT diagnosis for individual breed types over the study period was calculated from the number of MCT cases of a specific breed type divided by the population of dogs of the same breed type in the VetCompass system presenting to participating practices during the study period. A nested case–control study design identified risk factors associated with MCT diagnosis.

### Data analysis

Before importation into IBM SPSS version 20 statistical software for analysis [36], data were imported into Microsoft Excel 2010 to be cleaned and to produce one record per dog. Dates of confirmation of MCT diagnosis for cases, and for controls dates of first recorded presentation to the veterinarian were used in combination with dates of birth to calculate ages. Insurance status on dates that directly preceded the first date of confirmed diagnosis was used for cases and insurance status nearest the first presentation was used for controls. Weights used were those documented nearest the date of diagnosis confirmation for cases and first presentation for controls. Breed type was recorded at the originating practices based on consensus between the practice and owners. The breed types recorded were mapped to the specific breed terms within the VeNom coding system during the analysis and were reduced into 15 categories. These categories derived from the top 10 most common case breed types and top 10 most common control breed types along with a ‘other specific breed type’ and a ‘crossbred’ category, defined as dogs of mixed breed. ‘Crossbred’ dogs were retained as a separate group and used as the comparator group for the breed-type analyses because of a prevailing opinion that health characteristic may vary between dogs of specific breed types and crossbred dogs due to a higher degree of homozygosity within the breed types [14, 37, 38]. Crossbreds included all dogs that were not classified as breed types regardless of the suspected number of progenitor breeds in the parentage and were used as the comparator group based on their suspected higher general degree of heterozygosity. Further separation of crossbreds into those dogs with just two specific parental breed types and those with more mixed ancestry was not possible at this stage.

Univariable analysis assessed associations between risk factors and a diagnosis of MCT using Chi-squared and Mann–Whitney U-test for categorical and quantitative data respectively because data were non-normally distributed. Broadly significant variables from the univariable analysis (P ≤0.25) were taken forward for consideration in the multivariable logistic regression model [18]. Collinearity of variables taken forward was explored via standard statistical methods [39]. A manual forward selection step-wise construction method was taken for model building with statistical significance set at the 5% level. The forward step-wise regression used the likelihood ratio test. Clustering at the clinic level was evaluated with the addition of clinic ID as a random effect. Final variables were evaluated for pairwise interactions and the final model was evaluated with the Hosmer-Lemeshow goodness-of-fit test [40].

## Abbreviations

CI: Confidence interval
EPR: Electronic patient record
MCT: Mast cell tumour
VeNom: Veterinary nomenclature.
